# Supplementary material for: Computational Prediction of Resistance Induced Alanine-Mutation in ATP Site of Epidermal Growth Factor Receptor
Source: Int J Mol Sci. 2022 Dec 13;23(24):15828. doi: 10.3390/ijms232415828 (PMC9784575; doi:10.3390/ijms232415828)
Supplement: Supplementary file 1 [file ijms-23-15828-s001.zip › ijms-1990636-supplementary.pdf]

Supplementary Materials

Computational Prediction of Resistance Induced Alanine-Mutation in ATP Site of Epidermal Growth Factor Receptor

Tasia Amelia [1], Aderian Novito Setiawan [1], Rahmana Emran Kartasasmita [1], Tomohiko Ohwada [2] and Daryono Hadi Tjahjono [1,*]

[1] School of Pharmacy, Bandung Institute of Technology, Jalan Ganesha 10, Bandung 40132, Indonesia
[2] Graduate School of Pharmaceutical Sciences, The University of Tokyo, Tokyo 113-0033, Japan
* Correspondence: daryonohadi@fa.itb.ac.id; Tel.: +62-22-250-4852

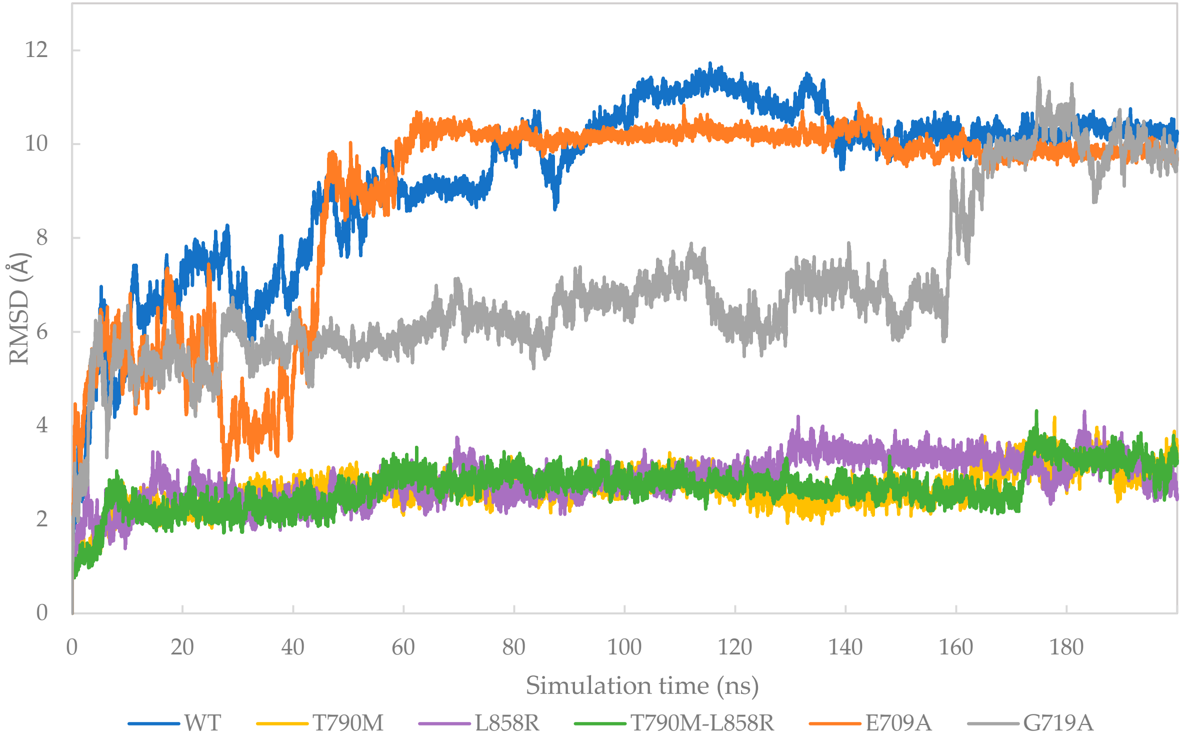

Figure S1. RMSD graph of references mutated receptor-ATP complex

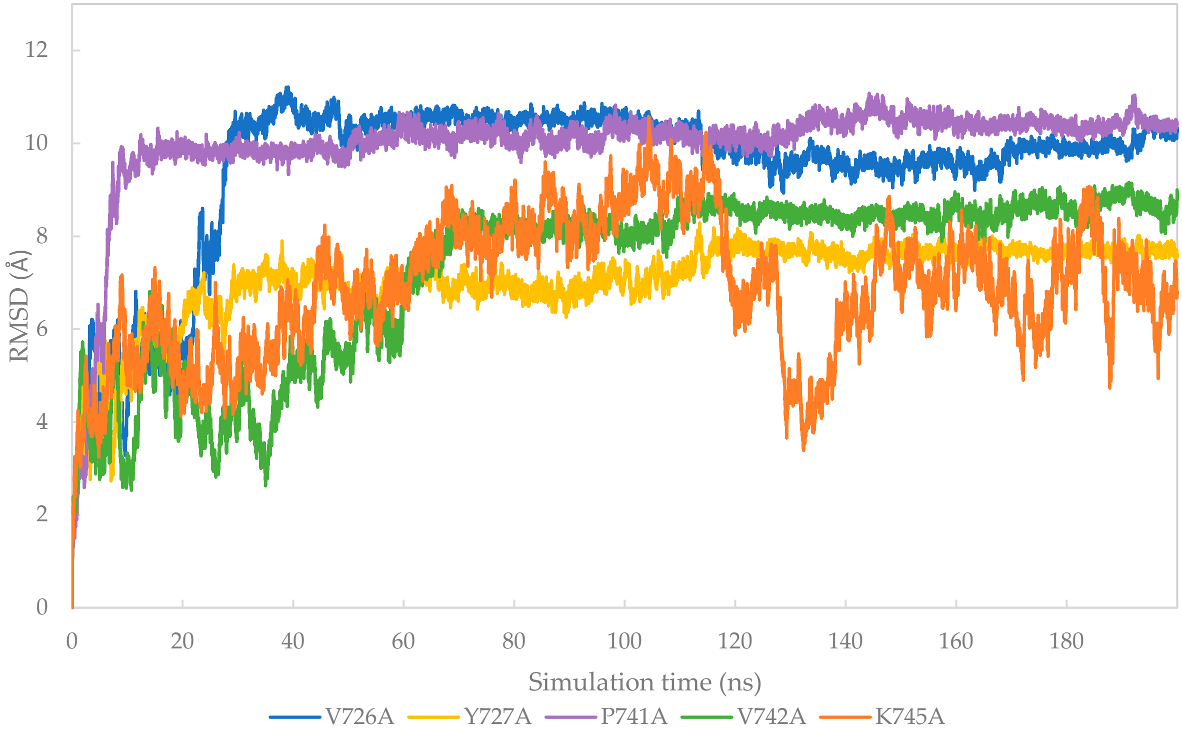

Figure S2. RMSD graph of V726A, Y727A, P741A, V742A, and K745A against ATP

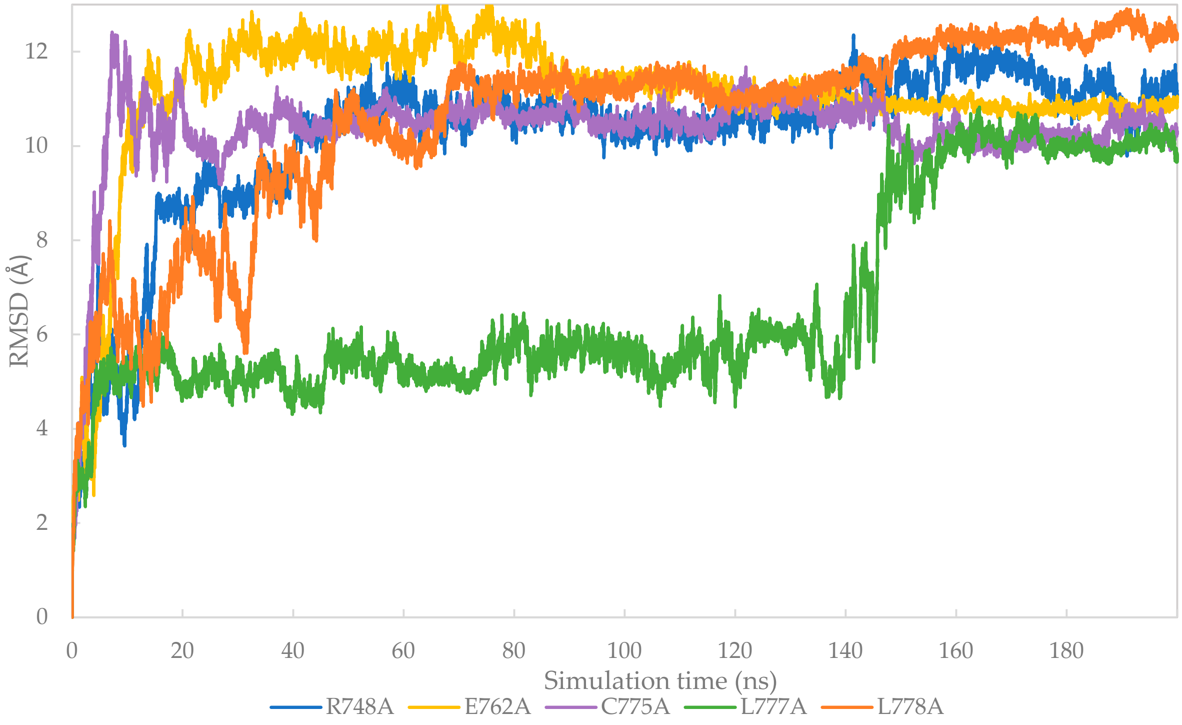

Figure S3. RMSD graph of R748A, E762A, C775A, L777A, and L778A against ATP

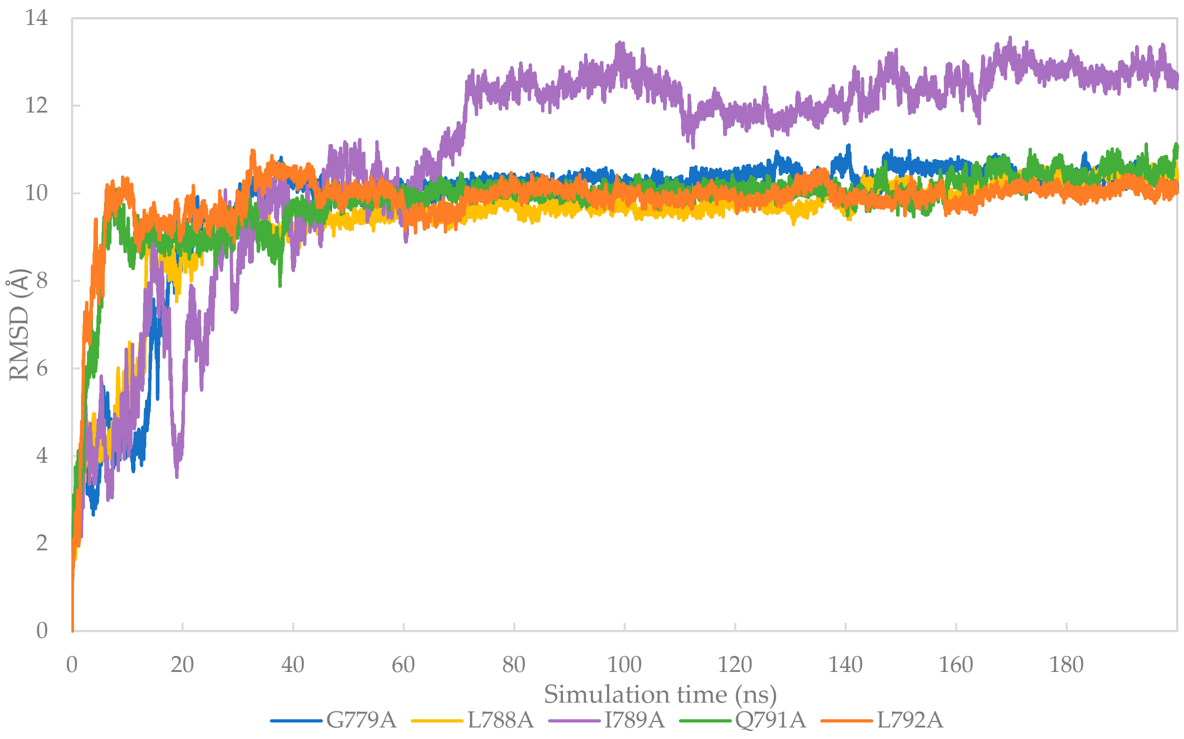

Figure S4. RMSD graph of G779A, L788A, I789A, Q791A, and L792 against ATP

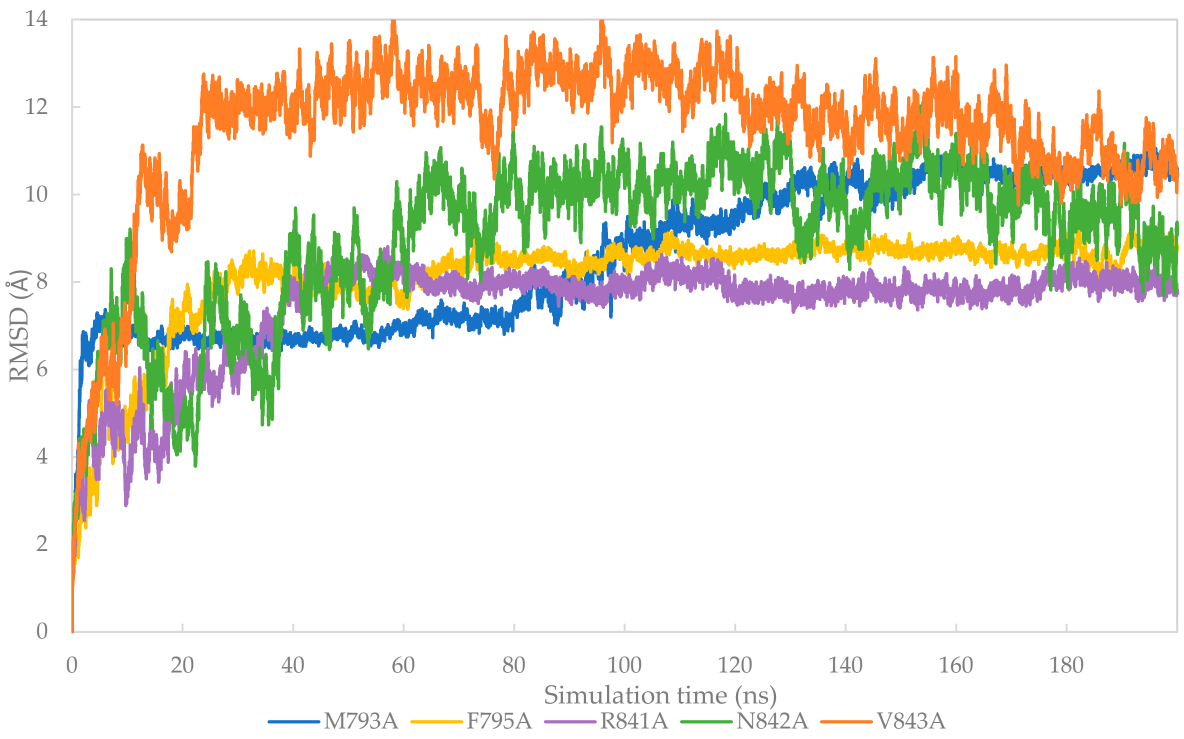

Figure S5. RMSD graph of M793A, F795A, R841A, N842A, and V843A against ATP

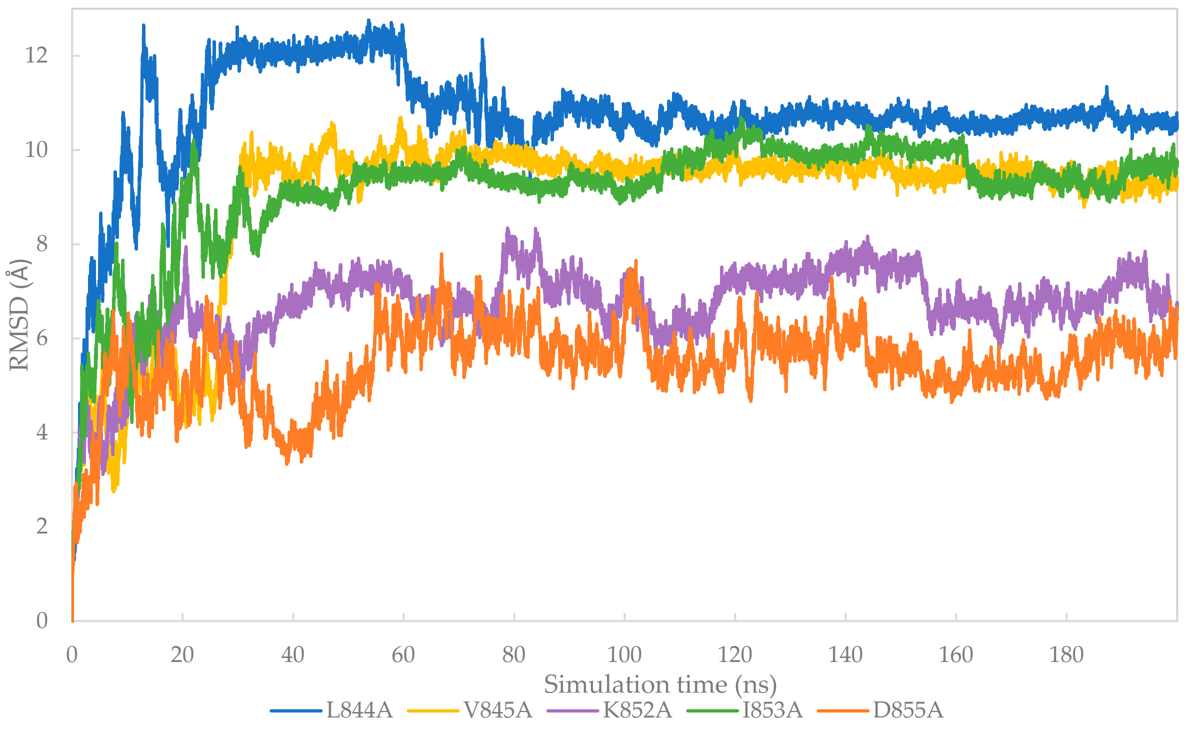

Figure S6. RMSD graph of L844A, V845A, K852A, I853A, and D855A against ATP

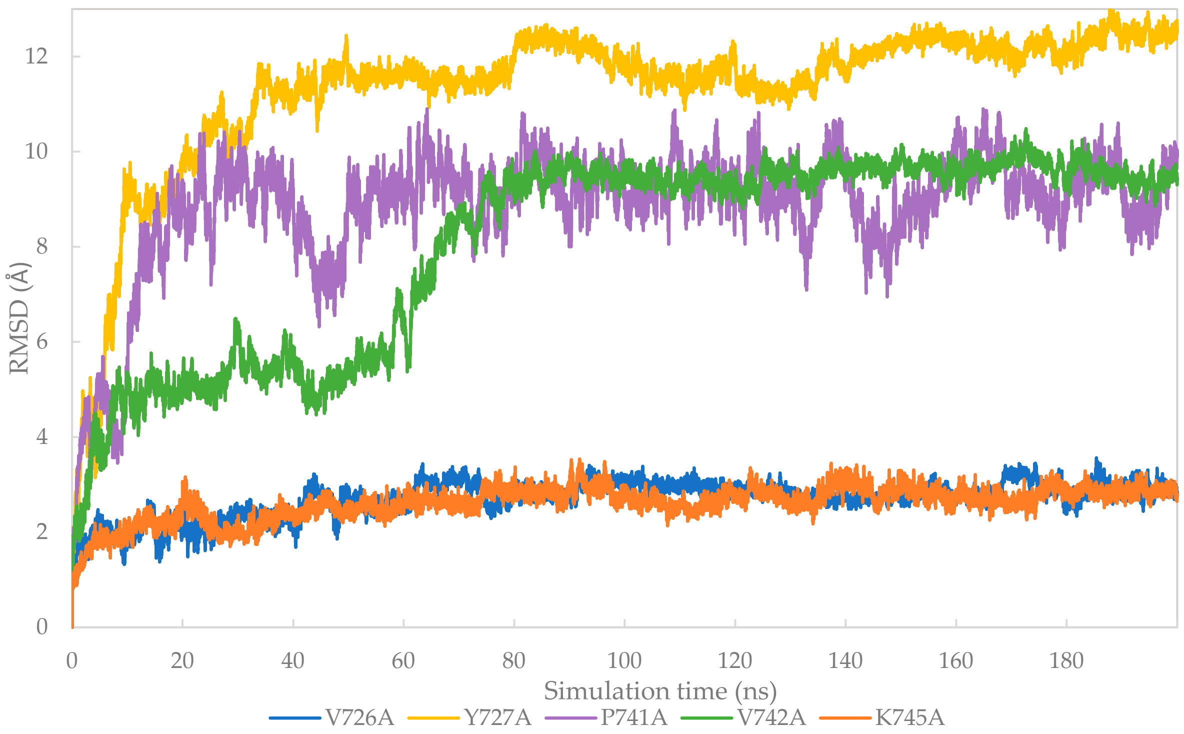

Figure S7. RMSD graph of V726A, Y727A, P741A, V742A, and K745A against Erlotinib

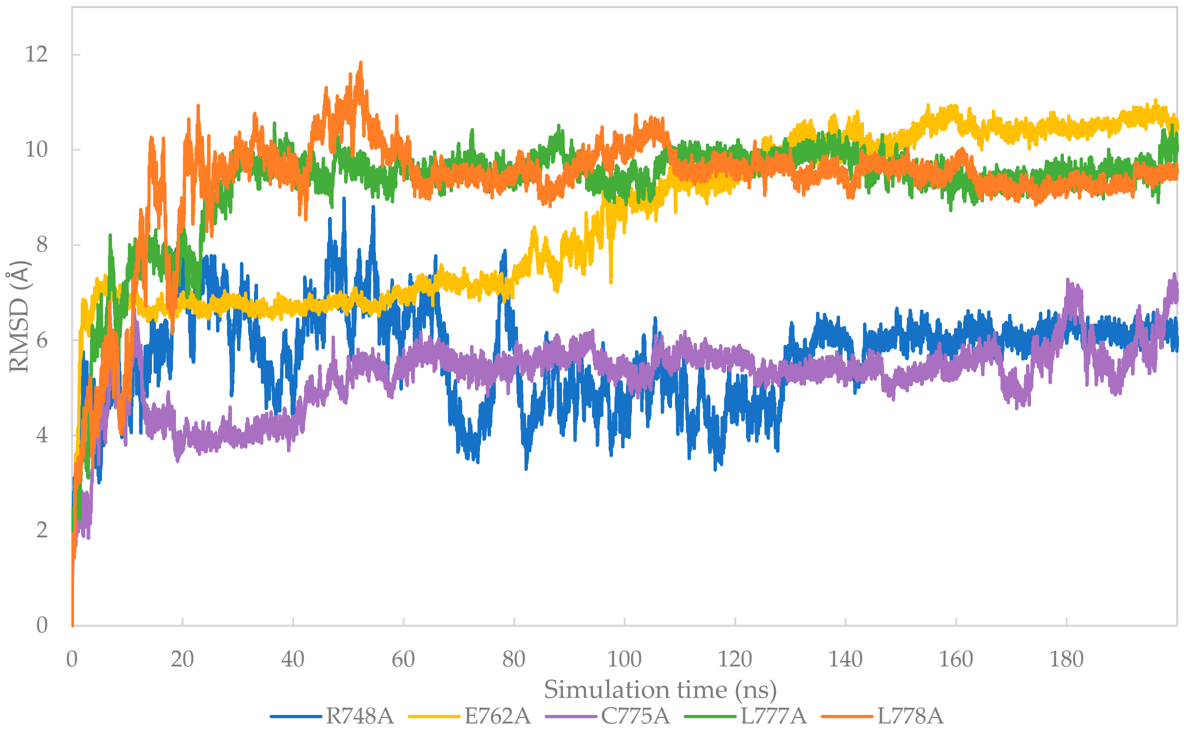

Figure S8. RMSD graph of R748A, E762A, C775A, L777A, and L778A against Erlotinib

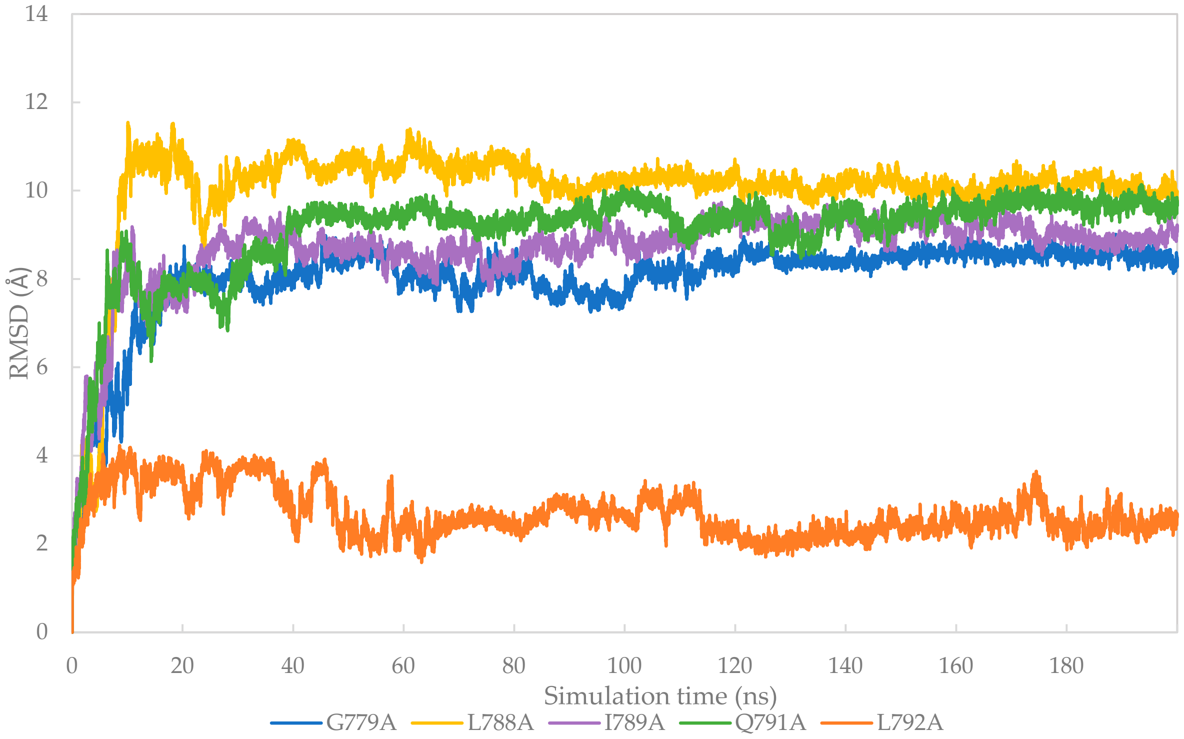

Figure S9. RMSD graph of G779A, L788A, I789A, Q791A, and L792 against Erlotinib

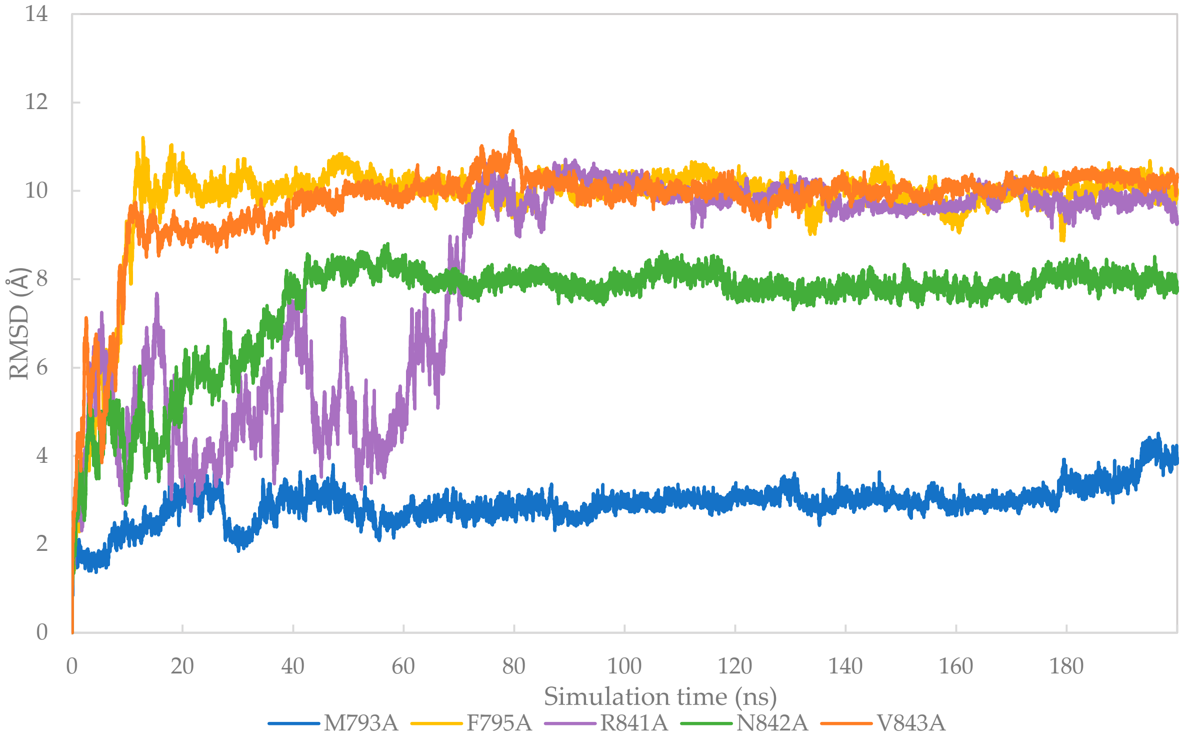

Figure S10. RMSD graph of M793A, F795A, R841A, N842A, and V843A against Erlotinib

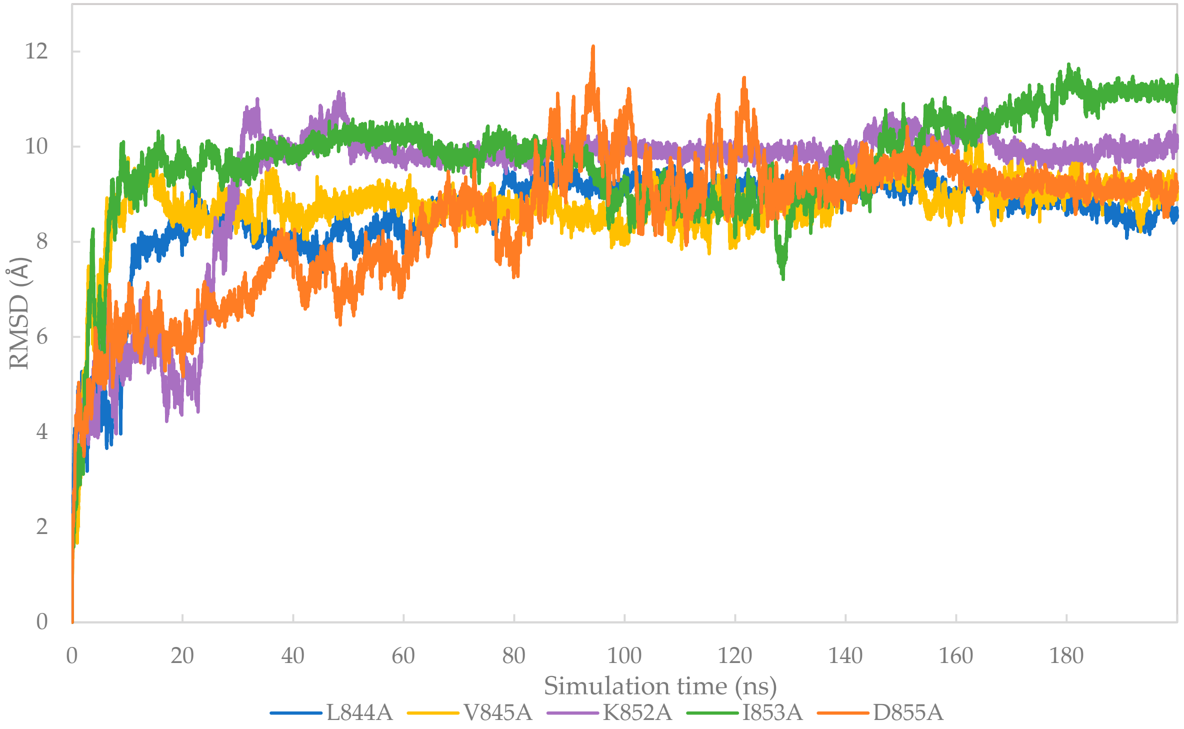

Figure S11. RMSD graph of L844A, V845A, K852A, I853A, and D855A against Erlotinib
